# Lessons Learnt from COVID-19: Computational Strategies for Facing Present and Future Pandemics

**DOI:** 10.3390/ijms24054401

**Published:** 2023-02-23

**Authors:** Matteo Pavan, Stefano Moro

**Affiliations:** Molecular Modeling Section (MMS), Department of Pharmaceutical and Pharmacological Sciences, University of Padova, Via Marzolo 5, 35131 Padova, Italy

**Keywords:** COVID-19, SARS-CoV-2, rational drug design, CADD, SBDD, homology modeling, docking, pharmacophore, protein–ligand interaction fingerprints, molecular dynamics

## Abstract

Since its outbreak in December 2019, the COVID-19 pandemic has caused the death of more than 6.5 million people around the world. The high transmissibility of its causative agent, the SARS-CoV-2 virus, coupled with its potentially lethal outcome, provoked a profound global economic and social crisis. The urgency of finding suitable pharmacological tools to tame the pandemic shed light on the ever-increasing importance of computer simulations in rationalizing and speeding up the design of new drugs, further stressing the need for developing quick and reliable methods to identify novel active molecules and characterize their mechanism of action. In the present work, we aim at providing the reader with a general overview of the COVID-19 pandemic, discussing the hallmarks in its management, from the initial attempts at drug repurposing to the commercialization of Paxlovid, the first orally available COVID-19 drug. Furthermore, we analyze and discuss the role of computer-aided drug discovery (CADD) techniques, especially those that fall in the structure-based drug design (SBDD) category, in facing present and future pandemics, by showcasing several successful examples of drug discovery campaigns where commonly used methods such as docking and molecular dynamics have been employed in the rational design of effective therapeutic entities against COVID-19.

## 1. The COVID-19 Pandemic

In December 2019, a cluster of pneumonia cases of unknown etiology emerged in the Chinese city of Wuhan [1]. Soon after, analyses of patients’ lung fluid, blood, and throat swabs reconducted this outbreak to a newly identified virus, tentatively named 2019-new coronavirus (2019-nCoV) [2].

Phylogenetic analyses performed on viral genomes isolated from patients’ samples revealed a close relationship between the new virus with several bat coronaviruses isolated in China (>90%). A lower degree of similarity was also found with SARS-CoV (80%) and MERS-CoV (50%), the causative agents of two recent coronavirus-related epidemics [3]. Based on phylogeny, taxonomy, and established practice, the virus was renamed SARS-CoV-2 [4], while the associated illness was defined as COVID-19 by the World Health Organization (WHO) [5]. 

The striking similarity between the SARS-CoV-2 genome and several bat coronaviruses led to the hypothesis that bats could be the animal reservoir for SARS-CoV-2, with pangolins or other mammals acting as the intermediate host before human transmission [6]. The assumption that bats could be the animal reservoir of SARS-CoV-2 was further reaffirmed at a later stage by the work of Temmam et al., which identified in the caverns of North Laos a series of bat coronaviruses that share a high level of sequence similarity (96%) with the SARS-CoV-2 genome [7]. 

From a clinical perspective, the spectrum of COVID-19 manifestation is broad, ranging from asymptomatic infections to severe viral pneumonia with respiratory failure and even death [8]. The most common symptoms, similar to influenza, are related to mild upper respiratory tract affection, such as fever, cough, myalgia, and headache [9]. Less common but still relevant ones include gastrointestinal manifestations, such as diarrhea, and more severe respiratory illnesses, such as dyspnea, and multiorgan failure [10]. 

The long incubation time compared to similar infections [11], the capability of asymptomatic [12] or paucisymptomatic [13] patients to transmit the virus even before the eventual symptoms’ manifestation, and the aerial transmission modality [14,15] all concurred to determine a higher transmissibility index (estimated between 2.5 and 3.0) for the SARS-CoV-2 virus, compared to similar viral infections [16]. These factors contributed to the rapid spread of SARS-CoV-2 worldwide, resulting in more than 650 million cases and more than 6.5 million deaths globally [17]. 

In the first stages of the COVID-19 pandemic, extraordinary sanitary measures, such as physical and social distancing, wearing face masks, and eye protection devices [18,19] were adopted to prevent the collapse of the public healthcare system [20], due to the imbalance between the high demand and the low availability of critical supplies [21,22]. Although this short-term plan has proven helpful in gaining time [23,24], more sustainable and long-term oriented strategies were needed to better cope with the socio-economic [25] and psychological [26] consequences of the pandemic, other than ensuring fair and efficient resource management [27].

### 1.1. Drug Repurposing

Considering that bringing a brand-new drug on the market is usually a very long and expensive process [28], the so-called “drug repurposing” was the first approach to finding suitable therapeutic options for COVID-19 patients [29,30]. This strategy extends the applicability domain of already marketed drugs for treating diseases other than the one it was conceived for [31]. This approach is appealing because it involves using derisked compounds, with potentially lower overall development costs and shorter development timelines [32]. Unfortunately, despite all the promising premises [33], this approach was largely unsuccessful [34]. Indeed, several investigated drugs showed little to no efficacy in randomized clinical trials [34]. The few successful cases were primarily symptomatic treatments, mostly limited to hospital usage for the most severe cases due to the therapy’s high costs or route of administration [35]. 

Failure of the drug repurposing strategy against COVID-19 can be mostly traced to the very first stages of the pandemic, where few clinical pieces of evidence were available for the rational elaboration of therapy plans. For example, the combination of HIV protease inhibitors Lopinavir and Ritonavir was examined [36], despite a suboptimal predicted recognition pattern towards the SARS-CoV-2 main protease (M^pro^) compared to other compounds of the same class [37]. Another example is the combined use of an antimalaria drug (hydroxychloroquine) and an antibiotic (azithromycin) despite no clear indication of the possible mechanism of action [38,39]. 

With more and more clinical observations becoming available, more fine-tuned treatments, especially symptomatologic ones, were adopted. This is the case, for example, of corticosteroids such as dexamethasone [40], employed to tame the inflammatory response associated with severe COVID-19 cases, and low molecular weight heparins [41], used to prevent or treat thrombo-embolic events associated caused by interference with the cardiocirculatory system. 

A group of antiarthritis drugs represents another successful example of drug repurposing to their ability to modulate the immune response [42] and cytokine storm [43] caused by severe SARS-CoV-2 infection. This family includes the monoclonal antibodies Tocilizumab [44] and Sarilumab [45], which both inhibit Interleukin-6 (IL-6) signaling; Anakinra [46], which interferes instead with IL-1 signaling; and the Janus Kinase (JAK) inhibitor Baricitinib [47], alone or in conjunction with Remdesivir [48], with the latest representing maybe the most successful example of drug repurposing against COVID-19 being the first approved drug against this illness [49].

Originally designed against Ebola virus, Remdesivir is a nucleotide analog prodrug that acts as a viral polymerase inhibitor [50] and is efficient in shortening the recovery time in hospitalized adult patients affected by COVID-19 [51]. Unfortunately, as previously mentioned, Remdesivir and the other repurposed drugs need parenteral administration, thereby limiting their massive-scale adoption as pharmacological treatments against COVID-19 [35]. 

### 1.2. Convalescent Plasma and Monoclonal Antibodies

With the first round of spontaneously healed patients, doctors started flanking standard treatment with the use of convalescent plasma (CP), i.e., the plasma derived from recently recovered donors with a sufficiently high neutralizing antibody titer [52]. A similar protocol was previously adopted to face Ebola [53] and MERS [54] outbreaks, justifying its emergency use in the first stages of the COVID-19 pandemic. Unfortunately, despite promising observational data from the first studies performed on small-size patient cohorts [55], more thorough investigations from more extensive clinical trials demonstrated the inefficacy of this treatment [56,57], leading to its dismission from routine clinical practices. Despite this failure, CP inspired the design of safer and more targeted immunological treatments in the form of monoclonal antibodies (mAbs) [58,59]. Since the beginning of the pandemic, several mAbs directed against COVID-19 have been developed, with some obtaining approval from regulatory agencies [60]. Multiple of these mAbs are often used in conjunction to combine their neutralizing power and boost their therapeutic efficiency, exploiting their ability to bind at different epitopes [61].

The list of approved ones contains the therapeutic combinations of casirivimab and imdevimab (Regeneron/Roche), redanvimab (Celltrion Healthcare), sotrovimab (GSK), and the combination of tixagevimab and cilgavimab [62,63]. Furthermore, the association of bamlanivimab and etesevimab is nearly approved, despite the previous failure of trials investigating bamlanivimab on its own [63]. 

### 1.3. Vaccines

As seen in the case of CP and mAbs, a targeted immune response against SARS-CoV-2 can be a beneficial treatment for patients [64]. While immunoglobulins are limited to treating ongoing infections in hospital settings due to the high costs and the parenteral administration route, a more economical and scalable approach would be instructing the human body to produce this type of response without needing external intervention [65]. Based on this assumption and parallel to the drug repurposing approach, the industry and academia spent a consistent joint effort on developing preventive tools to avoid the infection in the first place or at least mitigate the most detrimental effects of the illness. This endeavor resulted in the quick approval by regulatory agencies of several vaccines [66]. 

Three different classes of these therapeutic entities can be recognized [67]. The first one, related to inactivated virus vaccines, comprises the Chinese CoronaVac (Sinovac) and the Russian CoviVac. The second group is formed by adenovirus vector vaccines such as Vaxzevria/ChAdOx1-S (AstraZeneca), Sputnik V/Gam-COVID-Vac, and Jcovden/Ad26.COV2.S (Janssen). Finally, the third one is composed of mRNA-based vaccines, including Comirnaty/BNT162b2 (Pfizer-BioNTech) and Spikevax/mRNA-1273 (Moderna) [68,69]. 

Despite the poor performances of the first class of vaccines [70,71], several independent studies have asserted worldwide the efficacy of vaccination campaigns based on the other two types of vaccines, particularly in the case of mRNA-based ones [72,73]. 

### 1.4. Spike Protein

The ability of the SARS-CoV-2 virus to infect human cells heavily depends on a surface glycoprotein known as the S/spike protein [74], named after its peculiar shape [75]. For this reason, both mRNA vaccines and mAbs are designed to target this protein and prevent the virus’s entry into the cell, thereby limiting its replication [76]. 

Concerning these, although different pathways for SARS-CoV-2 cell entry are possible [77,78], the principal and better-characterized one involves binding to the human ACE2 receptor (hACE2) [79], a membrane-anchored metallopeptidase that is abundantly present in various districts of the human body, from the vascular endothelium to the epithelia of lungs and small intestine [80]. On its own, host cell receptor binding is not sufficient to ensure entrance within host cells. Priming and activating the S protein by host proteases is required to enhance its cell–cell and virus–cell fusion processes and increase viral shielding from neutralizing antibodies [79,81]. The list of priming proteases includes, but is not limited to, TMPRSS2, a transmembrane serine protease that is often co-expressed with ACE2 in SARS-CoV-2 target cells; Furin; and cathepsin B/L [79,82,83]. The priming process entails the exposure of a lipophilic fusion peptide (FP), which penetrates the host cell membrane triggering the viral fusion [84] thanks to its strong membrane-perturbing capacities [85]

From a structural perspective, the spike is a trimeric transmembrane glycoprotein composed of 1273 amino acids organized in two main subunits, S1 and S2, and several functional domains [86]. 

**Figure 1:**
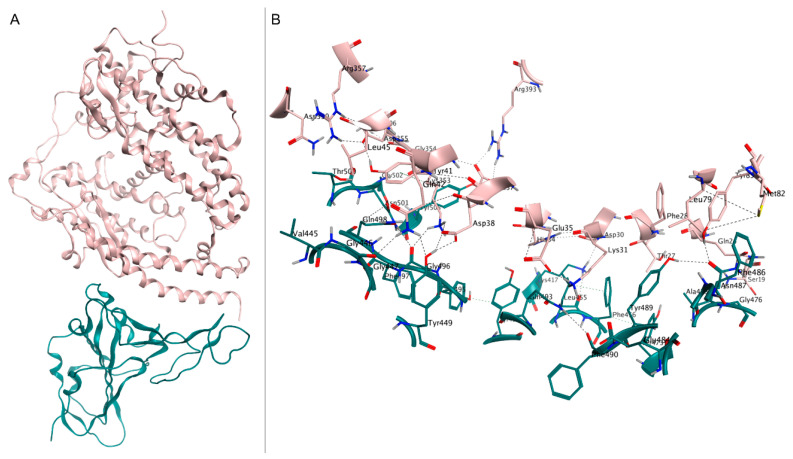
(**A**) Crystal structure of spike RBD (teal) in complex with hACE2 (pink), deposited in the Protein Data Bank with accession code 6M0J. (**B**) Close-up view of interface contacts between the spike RBD and hACE2: hydrogen bonds are represented as black dashed lines.

The S1 subunit comprises two main domains, specifically the N-terminal and C-terminal domains (NTD and CTD, respectively), which are both involved in the binding to host cell receptors [86]. The CTD contains the receptor-binding domain (RBD, residues 319–541), consisting of two motifs. Firstly, a core structure is formed by a twisted five-stranded antiparallel β sheet (β1, β2, β3, β4, and β7), with three short helices (α1, α2, and α3). Secondly, an extended loop (receptor binding motif, RBM) is formed by a two-stranded β sheet (β5 and β6), lying at one edge of the core and containing most of the residues involved in binding to hACE2 [87] (Figure 1).

The S2 subdomain has significant roles in spike protein trimerization and in mediating the virion entry into the host cell once the molecular contacts have been established [88]. It is formed by relevant subdomains such as the transmembrane domain (TD) (residues 1296–1317), which exerts both the spike anchoring to the outer side of the viral membrane and the maintenance of the trimeric quaternary structure [89,90], and a cytoplasm domain (CD) (residues 1318–1353), which mediates viral assembly and cell–cell fusion [91]. Furthermore, the previously mentioned fusion peptide, a cleavage S2′ site (residues 815/816), and two heptad-repeat domains (HR1/HR2) (residues 984–1104/1246–1295) are also part of S2 [92].

### 1.5. Viral Variants

Due to its exposition on the external surface of the SARS-CoV-2 membrane and its pivotal role in the virus’s ability to infect host cells, the spike protein is often subjected to mutations that alter the virus’s infectivity and antigenicity [93,94]. Therefore, since the spreading of the original viral strain (Wuhan-Hu-1) began, several viral variants appeared on the scene [95], particularly in third-world nations where collective sanitary practices such as social and physical distancing [96] or wearing face masks in public places [18] were hardly implementable [97].

The insurgence of novel viral strains with different susceptibility to the protective effect of vaccines [98] demands periodical updates of their original formulations coupled with multiple booster shots to maintain their efficacy [99], thus hampering the management of the pandemic based on massive vaccination of the world population [100,101].

Among the large pool of SARS-CoV-2 mutations [102], some gathered the scientific community’s attention due to their increased fitness, gaining the “variant of concern” (VOC) status [103].

The first ever SARS-CoV-2 VOC was the B.1.1.7 variant, more commonly referred to as the “Alpha” or “English” variant due to being first identified in November 2020 in the Kent region of the United Kingdom [104,105]. Despite worries about the higher transmissibility compared to other circulating variants at the time [106,107], clinical studies demonstrated how mAbs, CP, and especially vaccines, were still able to confer protection against B.1.1.7 [108,109,110], containing its impact on the sanitary system [111]. 

Unfortunately, soon after the emergence of the Alpha variant, a more threatening VOC arose. The B.1.617.2 variant, commonly known as the “Delta” or “Indian” variant, due to being first identified in India in late 2020, quickly overthrew B.1.1.7 thanks to its strikingly increased transmissibility [105]. The advent of the Delta variant was associated with the first signs of reduced protection provided by mAbs, CP, and most importantly, vaccines [112,113,114], thanks to its increased immune system evasion capability [115], posing a heavier workload on the sanitary system [116].

The latest hallmark in the history of SARS-CoV-2 variants is represented by the B.1.1.529 variant, first detected in South Africa and more often called the Omicron variant [117]. The combination of increased transmissibility [118] and immune system evasion [119] conferred this variant a net selective advantage in bypassing the protection provided by the complete primary vaccination cycle and a variety of clinically utilized mAbs [120,121,122] compared to other circulating strains. The ground-breaking impact the Omicron variant had on the worldwide spread of SARS-CoV-2 even led to the introduction of the “booster dose” to compensate for the reduced coverage of the primary vaccine cycle [98,123].

Lately, several subvariants germinated from the original Omicron strain (also labeled as BA.1), namely BA.2, BA.3, BA.4, and BA.5 [124,125,126]. Although different studies indicated how the first identified Omicron subvariants (BA.2 and BA.3) were similarly susceptible to existing treatments despite their increased transmissibility [127,128,129], it also emerged how the most recently identified ones (BA.4 and BA.5) are significantly more efficient in evading the immune response [130,131,132].

These findings indicate that SARS-CoV-2 continued to evolve by increasing its immune-evasion capability rather than counting on sheer higher transmissibility, sustaining the virus spread even in populations with high vaccination frequency and recovery rates [130,131,132]. 

### 1.6. Main Protease (3CL^pro^)

Considering the uncertainty about the efficacy of existing treatments [133] and booster vaccinations [134] against present and future Omicron subvariants, the need to find more reliable and variant-agnostic therapeutic tools against COVID-19 is emerging. The previously mentioned issues with the continuously mutating spike protein, which affects most present gold-standard COVID-19 treatments, indicate that different viral targets should be explored for developing novel antiviral drugs [135]. Generally speaking, an ideal target would have to play a pivotal role in the virus replication cycle and be highly conserved across different viral strains [136]. Within SARS-CoV-2, this role is portrayed by its main protease [137] (M^pro^, or 3C-like protease / 3CL^pro^ due to similarities with the picornavirus 3C protease [138]), thanks to its conserved fold across different coronaviruses [138,139,140,141] (including SARS-CoV [142]) and essentiality for the replication of this virus’s subfamily [143]. 

SARS-CoV-2 M^pro^, also called nsp5, is a cysteine protease composed of 306 residues [144] that steers the maturation of two partially overlapping polyproteins (pp1a and pp1ab) into individual mature nonstructural proteins (including M^pro^ itself) through their proteolytic cleavage [145].

Functionally speaking, M^pro^ exists in equilibrium between a monomeric and a homodimer form [146,147,148]. This dimerization directly influences the shape of the catalytic site [147], thus altering the enzymatic activity [138] and playing an indirect regulatory role during the virus replication cycle [149,150]. 

Within the M^pro^ functional dimer, each protomer is composed of three structural domains. The chymotrypsin-like fold, including β-barrel domain I (residues 1–99) and II (residues 100–182), hosts the active site and thus has direct control over the catalytic event [138,147], while the α-helical domain III (residues 198–306) is mainly involved in the direct regulation of dimerization, exerting only a secondary and indirect role on regulating M^pro^’s enzymatic activity [151]. Between the second and third domains lies a flexible 16-residue loop (residues 183–197) [152]. 

**Figure 2:**
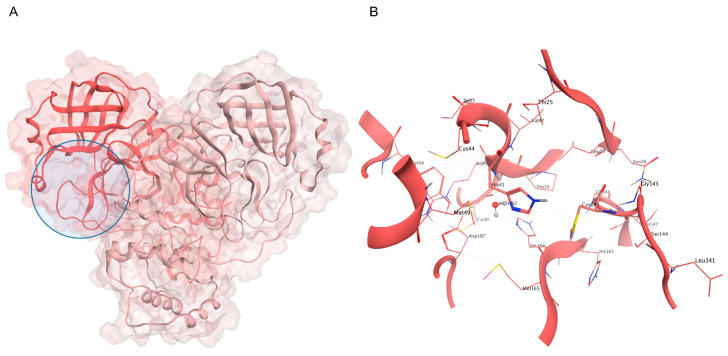
(**A**) Crystal structure of SARS-CoV-2 M^pro^ (PDB ID: 6Y2E): the first protomer is colored in salmon, while the second protomer is colored in pink, and the active site position is highlighted with a blue circle. (**B**) Close-up view of the catalytic site: the H41-C145 dyad is highlighted, alongside the conserved water molecule that substitutes the third member of the canonical catalytic triad diffused in several cysteine proteases.

As anticipated, the catalytic site is located between domains I and II, bordered by the N-terminal domain I of the second protomer in the dimer (Figure 2). Notably, the N-finger (residues 1–7) interacts with the binding site through a salt bridge between the positively charged end of Ser1 and the negatively charged end of Glu166 [153]. The latter is also involved in forming a hydrogen bond with His172, an essential interaction for the enzyme’s proteolytic activity [154]. These interactions are so crucial in stabilizing the catalytic site [155] that N-finger deletion impairs dimerization and abolishes the protease’s enzymatic activity [156]. 

M^pro^’s shallow, plastic, and solvent-exposed active site [152,157] comprises several subpockets (ranging from S6 to S3′), hosting the corresponding substrate residues (which vary from P6 to P3′) [139]. Speaking of substrates, the SARS-CoV-2 M^pro^ cleaves peptide bonds at the C-terminus end of a glutamine residue (P1) [137], which is conserved across different SARS-CoV-2, SARS-CoV, and even MERS-CoV substrate sequences [152]. 

SARS-CoV-2 M^pro^ recognizes sequences as long as ten residues (P6–P5–P4–P3–P2–P1↓P1′–P2′–P3′ P4′, where ↓ indicates the scissile bond [139]), but only shows remarkable selectivity at four subsites: S4, S2, S1, and S1′ [158]. On the contrary, prime recognition subsites located at the C-terminus of the conserved P2 (Leu/Val/Phe), P1 (Gln) ↓-P1′ (Ser/Ala) sequence are not conserved and show remarkable plasticity [152,159]. Furthermore, the main structural alterations of the binding site derive from flexibility at residues that line the S1 subpocket and segments incorporating methionine 49 and glutamine 189 [152,160]. 

Different from many other chymotrypsin-like proteases, M^pro^ exerts its enzymatic functions through a catalytic dyad instead of the usual triad, where His41 and Cys145 are flanked by a conserved water molecule that substitutes the sidechain of the third component (usually an aspartate or an asparagine) [138,161]. 

Aside from the catalytic dyad, another vital component of the catalytic machinery is represented by a set of conserved residues contouring the S1 subpocket known as the oxyanion loop (138–145) [152,162]. Notably, the correct conformation [87,163,164] of the oxyanion hole (Gly143-Ser144-Cys145) is required for stabilizing the tetrahedral transition state through a coordinated series of hydrogen bonds involving the backbone amides [138,155,165]. Accordingly, alternative oxyanion loop conformations are associated with catalytically incompetent/inactive proteases [140,152,154,166,167]. 

### 1.7. Rational Design of COVID-19 Drugs

Several characteristics of the viral proteases family, including SARS-CoV-2 M^pro^, make them an attractive target for the rational development of tailored drugs against COVID-19. First, the low sequence identity with human proteases coupled with distinct cleavage-site specificities reduces the possibility of off-target/side effects associated with the therapy [168]. Second, the striking conservation of protein fold and structural organization of the active site among different members of the same family leads to the possibility of developing pan-coronaviral drugs [169]. Third, the abundance of structural data about the SARS-CoV-2 main protease (659 structures have been deposited in the Protein Data Bank [170] to date) makes it possible to exploit the state-of-the-art structure-based approaches in drug design [171]. Furthermore, a similar strategy has already proved successful in finding efficient treatments against the hepatitis C virus [172,173] and human immunodeficiency virus (HIV) [174,175]. Finally, the experience acquired studying the original SARS-CoV protease [176], in conjunction with the rapid release to the scientific community of the SARS-CoV-2 protease [164], certainly played a major role in determining its prominent place within most COVID-19 drug discovery campaigns. A detailed report on structural features of the 3CL^pro^ protease that can guide the design of novel inhibitors can be found in the work of Xiong et al. [177]. 

The first attempts at finding SARS-CoV-2 M^pro^ inhibitors involved the repurposing of existing protease inhibitors. Particularly, the hepatitis C protease inhibitor Boceprevir [178,179] and the feline coronavirus 3CL^pro^ inhibitor GC373 (derived from its prodrug GC376) [180] were found to be active in the low µM potency range against M^pro^ [181], with the latter being particularly interesting due its promiscuous anticoronaviral activity [182]. Both candidate drugs share a similar peptidomimetic scaffold, which entails the most prominent interaction features of the first identified ones [164]. 

Although these primary hit compounds present a good binding pattern, their evolution towards clinical candidates and drugs is prevented by two main factors: first, covalent inhibitors are usually associated with selectivity problems, due to their ability to react promiscuously with a plethora of nucleophile moieties [183]; second, the peptidomimetic scaffold is usually associated with suboptimal pharmacokinetic properties that affect the preferred route of administration [184]. 

In this regard, a step forward was obtained when the first SARS-CoV-2 M^pro^ inhibitors were able to reach clinical stage experimentation, namely PF-07304814 (lately renamed as Lufotrelvir), a prodrug for the active principle PF-00835231, and PF-07321332 (Nirmatrelvir). 

Lufotrelvir was originally developed by Pfizer in 2002–2003 for the SARS-CoV virus and later repurposed against the SARS-CoV-2 due to the high similarities between the two proteases [185]. Due to its efficacy against several viral strains in preclinical studies [186,187], it was advanced to the clinical stages of experimentation, albeit quickly overcome by Nirmatrelvir thanks to its more favorable pharmacokinetic profile [188]. 

**Figure 3:**
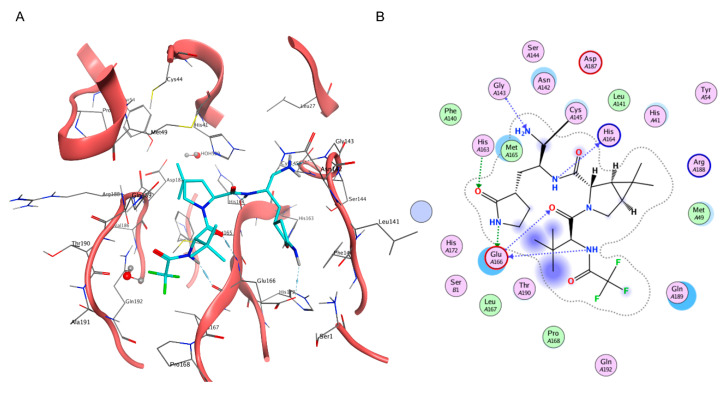
(**A**) Three-dimensional depiction of Nirmatrelvir orientation within the catalytic site of SARS-CoV-2 M^pro^ (PDB ID: 7RFW). (**B**) Bidimensional representation of intermolecular interactions of Nirmatrelvir–SARS-CoV-2 M^pro^ 7RFW complex.

Contrary to Lufotrelvir, which, similar to Remdesivir, requires parenteral administration, Nirmatrelvir can be administered orally [189], a must-have characteristic for the widespread adoption of drugs [190,191]. Designed by Pfizer amid the pandemic through the rational modification of Lufotrelvir [192], the structure of Nirmatrelvir was officially presented to the general audience on 6 April at the American Chemical Society Spring 2021 meeting [193], only one year after the official start of its development process [192] (Figure 3).

This peptidomimetic inhibitor, which is administered in association with the pharmacokinetic enhancer Ritonavir and sold under the commercial name of Paxlovid, represents a hallmark in the history of both the COVID-19 pandemic and structure-based drug discovery, due to the groundbreaking speed of its discovery campaign [194]. Although clinical studies highlighted the remarkable therapeutic efficacy of Paxlovid in preventing the most severe COVID-19 cases [195], its effectiveness on more mild infections remains unclear [196]. Furthermore, the impact of viral mutations on present and future protease inhibitors has yet to be disclosed [197,198], thus justifying the current effort to find novel and diverse drugs that can enlarge the pool of pharmacological tools available against COVID-19. 

An important step in this direction is represented by the development of Ensitrelvir (formerly known as S-217622), the first noncovalent, nonpeptidomimetic, orally available M^pro^ inhibitor to reach clinical stage experimentation [199]. This compound has successfully reached the third and final stage of clinical experimentation, thanks to its proven efficacy against mild-to-moderate or even asymptomatic infections [200,201]. Possible approval of this active principle by regulatory agencies would provide an additional and orthogonal therapeutic tool to Nirmatrelvir in the treatment of COVID-19 cases, thus reducing the impact of resistance mechanisms associated with the emergence of mutated viral strains [197,198]. 

### 1.8. Potential Targets of Interest

Although targeting the SARS-CoV-2 main protease was successful in individuating several clinical candidate drugs, even leading to the first approval of a COVID-19 specifically designed drug, other drug discovery campaigns aimed at different viral targets are needed for therapy diversification, potentially combined and synergic treatment, and resistance prevention [202,203,204]. 

Altogether, the SARS-CoV-2 genome encodes four major structural proteins, including nucleocapsid (N), membrane (M), envelope (E), and the spike as mentioned earlier (S), plus 16 nonstructural proteins, encompassing the previously mentioned main protease [205].

Although M^pro^ plays a pivotal role in processing the SARS-CoV-2 viral polyproteins, it is not the only component of the functional replicase complex that is required for the viral spread process [206]. Alongside this, a secondary but still relevant enzyme operates, namely the papain-like protease (PL^pro^, the catalytic domain of protein nsp3) [207]. Despite being a cysteine protease similar to M^pro^, PL^pro^ exerts its enzymatic functions through a catalytic triad composed of Cys111, His272, and Asp286 [208]. Further, PL^pro^ processes peptide bonds located at the C-terminal end of LXGG motifs [209]. Functionally speaking, this 343-residue segment, which is part of the multidomain nsp3 protein, is responsible for cleaving the SARS-CoV-2 polyproteins at three different sites, resulting in the liberation of nsp1, nsp2, and nsp3 proteins [210]. Moreover, PL^pro^ is also responsible for cleaving post-translational modifications on known regulators of host innate immune response [211]. 

As demonstrated by the approval of Remdesivir by regulatory agencies, another valuable target for the development of COVID-19 drugs is represented by the RNA-dependent RNA polymerase (RdRp) [49]. This complex machinery comprises four subunits, including one nsp12, responsible for the catalytic activity of the assembly; one nsp7; and two nsp8, with the latest two acting as cofactors [212]. The assembled holoenzyme presides RNA replication, a process that results in the formation of nine subgenomic RNAs [213]. The active site of nsp12 resides in its C-terminal RdRp domain and includes residues spanning from Thr611 to Met626, which are involved in binding one turn of double-stranded RNA, while residues D760 and D761 are required for recognition of the 3′ end and are essential for RNA synthesis [214,215]. Remdesivir binds within the active site, forming direct contact with residues K545, R553, D623, S682, T687, N691, S759, D760, and D761 and blocking the catalytic machinery by delaying the chain termination process [216,217]. 

During the RNA synthesis process, the RdRp also interoperates with nsp13 (helicase) [218], an enzyme involved in unwinding the RNA secondary structure of the 5′ untranslated section of the viral genome [219] to increase the efficiency of the copy process [220,221]. From a structural perspective, the nsp13 is a 596 residue, triangular pyramid-shaped helicase, which exploits its function thanks to the energy provided by its NTPase domain composed of six conserved residues (K288, S289, D374, E375, Q404, and R567) [222]. Adding to its helicase activity, the nsp13 active site also exerts RNA 5′ triphosphatase activity, further highlighting its importance in the maturation process of the viral mRNA [223]. 

The 5′ end of the newly synthetized mRNA is then subjected to post-translational modifications to boost both its stability (preventing cleavage from exonucleases), protein translation, and viral immune escape [224]. This activity is sequentially carried out by two S-adenosyl-L-methionine-dependent methyltransferases, namely nsp14 and nsp16 [225].

Specifically, the 527 residues’ nsp14 encompass both a proofreading exoribonuclease (ExoN) and an N7-methyltransferase enzymatic activity [226]. Furthermore, it has recently been suggested that it could encompass also a third, essential function for the viral replication cycle, based on the fact that SARS-CoV-2 ExoN knockout mutants are nonviable despite the 95% sequence identity with SARS-CoV [227] and the conservation of important active site amino acids including both the cap-binding residues (N306, C309, R310, W385, N386, N422, and F426) and the S-adenosyl methionine (SAM) binding residues (D352, Q354, F367, Y368, and W385) [228,229]. 

After its cleavage by the M^pro^, evidence suggests that it forms a binary complex with nsp10, which cooperatively exerts the proofreading activity on fresh RNAs produced by the RdRp machinery [230,231]. Although the binary complex theory is the most prominent one, an alternative hypothesis based on the formation of a ternary nsp10-nsp14-nsp16 has been proposed due to the flexibility of the lid subdomain of nsp14 and the fact that nsp10 also forms a heterocomplex with nsp16 [231].

Particularly, the nsp16-nsp10 heterodimer is responsible for the 2′ O-methyltransferase activity that is required to complete the cap-0 ➔ cap-1 conversion of mRNA that is initiated by nsp14 [225]. While the catalytic activity entirely resides on nsp16, nsp10 provides a support role, aiding the recruitment of both the m7GppA-RNA substrate (which happens at a binding site defined by residues K24, C25, L27, Y30, K46, Y132, K137, K170, T172, E173, H174, S201, and S202) and the SAM cofactor (which binds in a pocket defined by N43, G71, G73, G81, D99, D114, C115, D130, and M131), thus enhancing nsp16′s catalytic activity [232,233,234]. 

Lastly, another essential target for coronavirus biology is represented by nsp15, a uridine-specific endoribonuclease (NendoU) [235]. The active form of this enzyme is a dimer of trimers, with each monomer composed of 345 residues organized in three different domains: N-terminal, middle, and C-terminal NendoU, where the catalytic activity resides [236]. 

The active site contains six conserved residues: His250, His250, and Lys290, which compose the catalytic triad, and Thr341, Tyr343, and Ser294, with the latest associated with selectivity in substrate recognition [237]. Due to their localization within the hexamer, cooperativity or anticooperativity between different binding sites is possible [238]. Nsp15 enzymatic activity involves the cleavage of both single- and double-stranded RNA at uridine sites producing 2′,3′-cyclic phosphodiester, and 5′-hydroxyl termini [239]. 

Functionally speaking, Nsp15 seems to directly participate in viral replication through interference with the innate immune response [237]. Indeed, to evade host pattern recognition receptor MDA5 responsible for activating the host defenses, the Nsp15 cleaves the 5′-polyuridine tracts in (-) sense viral RNAs [240], though it has also been suggested that Nsp15 degrades viral RNA to hide it from the host defenses [238]. More detailed structural information about potentially druggable SARS-CoV-2 protein targets can be found in the works of Littler et al. [241] and Wu et al. [242].

## 2. Computer Simulations for Rational Drug Design

For most of its existence, the human genre has exploited natural products such as leaves, seeds, roots, bark, and flowers as medicines, based on empirical observations purely based on symptom relief [243,244]. 

Nevertheless, throughout the latest two centuries, the process of drug discovery has evolved rapidly from the serendipitous discovery of novel active principles derived from or inspired by natural compounds [245,246] to the rational design of brand-new chemical entities [247].

The major turning point in the history of modern drug discovery can be traced back to the 1980s when experimentally solved macromolecular structures become routinely available [248]. The enhanced accessibility of structural data about biological targets is reflected in a rapid interest in the development of computational methods that could valorize this information and aid medicinal chemists’ work [249]. 

Today, computer simulations are a staple point of drug discovery campaigns, thanks to their ability to streamline and reduce their attrition rate [250]. From a functional perspective, computer-aided drug discovery (CADD) techniques are employed in the earliest stages of the pipeline for hit identification, hit-to-lead optimization, and pharmacokinetic evaluations [251]. 

CADD methodologies can either fall into one of two subgroups, based on the rationale behind them: the first group is represented by ligand-based (LBDD) approaches, while the second one includes structure-based (SBDD) methods [252]. The main difference between these two orthogonal and complementary approaches is that the first one does not exploit any information about the target macromolecule structure (e.g., a protein or a nucleic acid), while the second one does [253]. 

Nowadays, with the advent of cryo-electron microscopy (cryo-EM) [254] and groundbreaking tools for de novo prediction of protein structures such as AlphaFold [255], the second approach has become the gold standard [171].

### 2.1. CADD Strategies against COVID-19

The starting point of every SBDD campaign is the identification of a target macromolecule (a protein or a nucleic acid) that is involved in the etiology and or pathogenesis of a disease of interest, whose function can be opportunely modulated through a specifically designed ligand, usually a small organic molecule [171]. 

Once the target has been identified, its structure must be retrieved, either through experimental methods such as X-Ray crystallography (XRC, the gold standard) [256], nuclear magnetic resonance (NMR) [257], and cryo-EM [258] or hypothesized through homology modeling or de novo prediction [259]. 

Homology modeling involves the use of a homologous protein with a high primary sequence identity with the target as a template for the construction of its three-dimensional model [260,261]. De novo prediction, instead, does not rely on any information about other proteins’ structures and outputs a structural hypothesis that is solely based on the primary sequence of the target of interest [262]. 

While the second approach has gained a lot of momentum during the last two years, thanks to its unprecedentedly high accuracy [263,264], the first one is still relevant in those cases where important structural rearrangements occur between different states of the target functional cycle, other than predicting ligand-bound conformations [265,266]. 

In the context of the COVID-19 pandemic, where the extraordinary effort promoted by the scientific community quickly made several experimentally determined structures available, the relevance of structural modeling was highlighted by the ability to keep up with the high mutation rate of the virus [135,207], other than providing a useful starting point for drug discovery campaigns for a target whose structure had yet to be elucidated [267,268]. For example, several studies were conducted to investigate the impact of mutations found in both the spike protein [135,269,270,271,272,273] and the main protease [135,198,274,275] of emerging strains on viral fitness and resistance to existing therapies. These studies showed that relatively inexpensive approaches such as homology modeling and positional scanning can be reliable tools to rationalize the origin of the virus [274,276,277,278], quickly track the evolution of the original strain [135,279,280], predict the impact of future possible mutations [270,272] and adjust existing therapeutics tools accordingly [198,281]. 

The huge amount of structural information available on several SARS-CoV-2 druggable targets was fertile terrain for various COVID-19 SBDD campaigns [282,283], both in academia and in industry, with the most effort aimed at hitting well-characterized and pivotal viral targets such as M^pro^ or spike [284,285].

A remarkable example is represented by the COVID Moonshot Consortium, a drug discovery campaign driven by a collaborative effort among different research groups across the world aimed at targeting the SARS-CoV-2 main protease. This project led to the advancement of novel noncovalent orally available nanomolar M^pro^ inhibitors to clinical stage experimentation [286]. 

### 2.2. The Swiss Knife of SBDD: Molecular Docking

Within every SBDD campaign, available information about the target structure is exploited to fetch molecules able to recognize it selectively and potently [287]. Usually, this involves the identification of molecules that have good steric and electrostatic complementarity with the active site [288]. Depending on the steric and volumetric features of the binding site, the ligand type can be chosen accordingly, with small organic molecules being a better solution for buried cavities [289] and peptides, aptamers, or antibodies a better one for larger, flatter, and solvent-exposed interaction surfaces [290]. 

To narrow down the list of potentially active molecules to experimentally test to a feasible number, and to avoid wasting resources on compounds that do not possess the appropriate features to interact with the target, most SBDD campaigns start with a virtual screening process (SBVS) [291]. The most widely and successfully adopted method for SBVS is molecular docking, a computational protocol developed in the 1980s by Kuntz et al. [292] for predicting the preferred orientation of a certain ligand within the active site of a receptor [293]. 

Each docking program has two major components, which cooperate to find the solution to the protein–ligand docking problem [294]. The first part is the search algorithm (SA), which explores the ligand degrees of freedom within a user-defined search space centered around the active site of the protein [295]. The SA generates several ligand conformations (poses) that are fed to the second element of the program, i.e., the scoring function (SF), which qualitatively evaluates subsisting protein–ligand interaction features [296]. 

In the context of the COVID-19 pandemic, docking was also the king of computational methods used for drug discovery, thanks to the combination of its accuracy [297] and rapidity, which allows it to virtually screen billions of compounds in just a few days [298,299,300]. 

For example, Corona et al. reported the discovery of four low micromolar nsp13 inhibitors through a virtual screening carried out with the LiGen [301] docking program on an in-house natural compounds library [302]. 

Kolarič et al. identified two micromolar SARS-CoV-2 cell-entry inhibitors that act by binding human neuropilin-1 (nrp-1) and preventing its interaction with the spike protein, by performing a virtual screening with the GOLD [303] program on a library of commercially available compounds [304]. 

Vatansever et al. performed a virtual screening based on the Autodock [305] program on a library of drugs approved by the Food and Drug Administration and by the European Medical Agency (EMA) to discover six micromolar M^pro^ inhibitors [306]. 

Kao et al. reported the discovery of three sub-micromolar, synergistic nsp1 inhibitors identified through two independently executed virtual screenings with ICM [307,308] and Vina [309] software on a library of FDA-approved drugs [310]. 

Zhang et al. identified 11 natural compound M^pro^ inhibitors active in the low micromolar range through a virtual screening purely based on the commercial software Glide [311], developed by Schrödinger [312]. Another strategic use of docking-based virtual screening based on the Glide program is portrayed by the work of Huff et al., which designed six mixed covalent and noncovalent nanomolar M^pro^ inhibitors [313]. Another Glide-based virtual screening performed by Liu et al. led to the repurposing of histone deacetylase (HDAC) inhibitors as SARS-CoV-2 cell entry inhibitors through allosteric modulation of ACE2 and alteration of its ability to recognize the spike protein [314]. 

Wang et al. used LibDock [315] to perform a virtual screening on a library composed of FDA-approved peptides, which led to the identification of a nanomolar SARS-CoV-2 cell entry inhibitor that exerts its effect by binding the human ACE2 receptor [316]. 

A remarkable result was obtained by Luttens et al., which identified eight M^pro^ inhibitors (including a nanomolar compound with pan coronaviral activity) by combining fragment-based drug design with ultralarge virtual screening based on the DOCK [292] program [317]. 

Welker et al. exploited the molecular docking pipeline of the LeadIT [318] program to repurpose previously identified SARS-CoV PL^pro^ inhibitors towards its SARS-CoV-2 homolog, demonstrating their activity on viral replication in cell-based assays [319]. 

Otava et al. utilized docking calculations with the GOLD [303] software to rationalize the structure–activity relationship of a series of rationally designed S-adenosyl-L-homocysteine derivatives, some of which showed inhibitory activity towards SARS-CoV-2 nsp14 in the low nanomolar potency range [320]. 

Similarly, Wang et al. exploited docking with Vina to rationalize the SAR of a series of rationally designed phenanthridine nucleocapsid protein (NPro) inhibitors, including two compounds showing low micromolar inhibitory activity [321]. 

### 2.3. Complementary Strategies to Address Docking Limitations

Although a very efficient and useful tool, molecular docking is rarely used on its own within SBDD campaigns and, indeed, is most often coupled with other methods to compensate for its weak points, such as neglecting receptor flexibility or the role of solvents [322], thus increasing the virtual screening success rate [323]. Another major limitation is represented by the poor ranking capabilities of classical scoring functions [324], which is the main cause of the high false positive rate of docking-based virtual screenings [325]. Indeed, in order to be universally applicable across different biological targets and computationally efficient enough to evaluate a large number of compounds, scoring functions have some limitations in the physical description of the binding event, which prevent any correlation between docking scores and experimentally determined affinity values [296]. Furthermore, little to no difference in score exists between top-ranking compounds derived from large virtual screening campaigns, making it practically impossible to distinguish active from inactive compounds solely based on the docking score [326]. For these reasons, each docking-based virtual screening cannot be blindly executed and fully automatized, and a careful setup of the experiment must be executed based on the available literature data and the knowledge of the target [326,327]. For COVID-19, the importance of this common-sense medicinal chemistry practice has been highlighted by the retrospective literature analysis provided by Llanos et al., which showcased the poor performances of structure-based virtual screenings solely based on ranking provided by docking scoring functions [323].

A possible solution to the limited physical description of the protein–ligand binding event of docking is to couple it with molecular dynamics (MD) simulations [294,328]. Molecular dynamics is a computational technique that allows investigating the time-dependent evolution of biological systems following the rules of molecular mechanics, i.e., determining the atomic trajectories by numerically solving Newton’s equation of motion, where forces between the particles and their potential energies are calculated according to molecular mechanical force fields [329]. Due to the heavy computational workload required to run these types of simulations, MD is rarely used for screening purposes, while it is more frequently exploited for the refinement of docking results, i.e., evaluating the pose stability or optimizing the protein–ligand complex geometry for a more accurate estimation of the free binding energy [330,331].

Regarding the pitfalls of the scoring component of docking programs, one possible strategy is to apply some form of knowledge-based filter upon docking results, in a similar fashion to what would happen if each pose were visually inspected [332]. For example, experimental information about critical protein–ligand interactions required for binding can be encoded within a pharmacophore filter or an interaction fingerprint, both of which can be used as constraints in the pose selection process [333]. In the case of pharmacophore filters, poses are filtered based on their ability to place a given functional group within a defined volume [334,335], while in the case of protein–ligand interaction fingerprint, the selection is usually based on the similarity between the reference and the query vector, representing the interaction features of the reference compound (a true active) and the investigated molecule respectively [336,337]. 

For instance, Wang et al. used a combination of structure-based pharmacophore screening, docking (both performed with the appropriate tools of the Molecular Operating Environment suite), and postdocking molecular dynamics refinement to identify a set of four sub-micromolar M^pro^ inhibitors among a database of in-house compounds [338]. 

The same protocol was successfully exploited by Tian et al. to identify four sub-micromolar PL^pro^ inhibitors in the same in-house library [339]. 

Furthermore, a slight variation of the protocol was also employed by Yin et al. to discover a noncovalent cyclic peptide that simultaneously inhibits both SARS-CoV-2 M^pro^ and nrp-1 with an activity in the low nanomolar range [340]. Within this scientific work, pharmacophore constraints were used for scoring peptide poses on M^pro^, while traditional docking scores were used for the nrp-1 screening. 

A remarkable joint computational work by Gossen et al. led to the molecular dynamics-driven design of a structure-based pharmacophore filter, which was then exploited to identify two nanomolar M^pro^ inhibitors among a library of publicly available compounds [341]. 

A similar approach was exploited by Hu et al., which exploited the combination between MD-based pharmacophore filtering, docking-based virtual screening within the Molecular Operating Environment suite, and MD-based postdocking refinement to identify micromolar SARS-CoV-2 cell entry inhibitors targeting the FP of the spike protein [342].

Jang et al. used protein–ligand interaction fingerprint similarity as a postdocking filter for their double virtual screening on both M^pro^ and RdRp with the Vina program to identify seven compounds inhibiting SARS-CoV-2 replication in cell-based assays among a library of approved drugs [343]. 

Due to the static nature of molecular docking, which does not consider receptor flexibility, the choice of the input structure is vital for the success rate of a virtual screening [344]. Although molecular dynamics can be a useful posterior refinement of poses, a wrong input conformation of the target macromolecule could prevent the sampling of native-like poses for active compounds, leading to a reduced hit-finding rate [345]. For this reason, multiple conformations of the same receptor derived from MD simulations or experimentally solved in different conditions can be used in parallel in a process defined as ensemble docking (ED) [346]. When this approach is used, docking calculations are independently run on each structure, with virtual hit compounds being identified either through consensus scoring or a consensus ranking approach [347,348]. In the case of consensus scoring, the docking score of the same molecule is averaged across the different virtual screenings, with the final ranking based on the consensus score [349]. Differently, consensus ranking involves the selection of top-ranking hit compounds across different virtual screenings, regardless of congruence between scores [350]. A consensus approach can also be utilized to rank molecules based on virtual screening executed on the same receptor structures with different docking protocols [351].

For example, Gimeno et al. applied a consensus scoring approach to three independently executed virtual screenings through Glide, FRED [352], and Vina software to identify two M^pro^ micromolar inhibitors within the Drugbank database, a library that includes all drugs approved by the Food and Drug Administration (FDA) [353]. 

Yang et al., instead, employed an ensemble docking approach with the Glide docking software to identify six M^pro^ inhibitors among a library of commercially available peptidomimetic compounds, two of which demonstrated sub-micromolar potency [354]. 

Rubio-Martinez et al. used a combination of ensemble docking based on QVina2 [355] and postdocking molecular dynamics refinement to identify five M^pro^ micromolar inhibitors within a library of commercially available natural compounds [356]. 

A mixture of the previous two approaches was exploited by Clyde et al. for their High-Throughput Virtual Screening (HTVS), based on both ensemble docking and consensus scoring between the FRED and Vina docking programs, that led to the discovery seven micromolar M^pro^ inhibitors among a set of commercially available compounds [357]. 

Further, a combination of consensus ranking among Autodock, Hybrid, and FlexX and postdocking molecular dynamics refinement was utilized by Glaab et al. to virtually screen a library of commercially available compounds and identify two micromolar M^pro^ inhibitors [358]. 

Similarly, Ghahremanpour et al. applied both consensus ranking among three independent virtual screenings performed with the Glide, Autodock, and Vina software and postdocking molecular dynamics refinement to identify 14 micromolar M^pro^ inhibitors within the Drugbank database [359]. 

Another possible solution to cope with inaccuracy in free binding energy determination by traditional scoring functions is to rescore docking poses using more computationally intensive and accurate methods such as Free Energy Perturbation (FEP) [360] or MMGBSA/MMPBSA [361]. The first approach relies on performing a series of alchemical transformations across a set of ligands that need to be evaluated. This conversion cycle allows calculating relative differences in the free binding energy that can be used for a more accurate ranking of hit compounds derived from a virtual screening [362]. The second approach relies instead on correcting the gas phase interaction energy calculated according to the molecular mechanics force field with a term accounting for the desolvation-free energy, where the polar component is estimated either by numerically solving the Poisson–Boltzmann equation (MMPBSA) or through the Generalized Born method (MMGBSA) [363].

Intriguingly, one of the hit compounds identified in the work of Ghahremanpour et al. was then used by Zhang et al. for the FEP-driven design of multiple nanomolar M^pro^ inhibitors [364]. 

A similar combination of Glide docking and FEP to determine the absolute binding free energy was also employed by Li et al. to identify 15 micromolar M^pro^ inhibitors within the Drugbank database [365]. The efficacy of FEP in estimating the binding energy of potential M^pro^ inhibitors was also highlighted by a retrospective study by Ngo et al. [366]. 

A multistep virtual screening involving semiflexible docking with Glide, Schrödinger induced-fit docking [367], MD-based postdocking refinement, and binding free energy estimation with the MMGBSA [368] protocol was exploited by Ibrahim et al. to identify one low micromolar nsp15 inhibitor [369]. 

**Figure 4:**
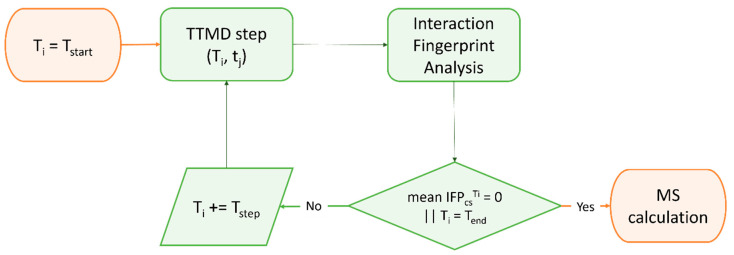
Workflow of a Thermal Titration Molecular Dynamics (TTMD) simulation. The time-dependent conservation of the native binding mode within a protein–ligand complex of interest is monitored with a scoring function based on interaction fingerprint through a series of short molecular dynamics simulations performed at progressively increasing temperatures. The simulation is carried out until the target temperature is reached or the dissociation process is completed. A coefficient called MS is then calculated and used to rank ligands based on the persistence of their native binding mode.

Although the estimation of thermodynamic properties such as the free binding energy has been a staple point of drug discovery campaigns, both from a computational and an experimental perspective, lately there has been a major interest shift towards the determination of kinetic parameters since they better correlate with in vivo efficacy [370]. Specifically, several MD-based methods have been developed throughout the years to rank compounds based on their predicted residence time, i.e., the time that the ligand spends in the receptor-bound state [371]. Among those, Pavan et al. developed Thermal Titration Molecular Dynamics (TTMD), a new method for qualitative estimation of protein–ligand complex stability (Figure 4), which was successfully applied for correctly discriminating tight, low nanomolar binders from weak, micromolar SARS-CoV-2 M^pro^ inhibitors [372]. 

### 2.4. Beyond Protein–Ligand Docking: Alternative Strategies for Rational Drug Development

Despite the indisputable relevance of molecular docking within most SARS-CoV-2 drug discovery campaigns, other approaches were successfully implemented, especially for projects which deviate from the design of a standard small molecule noncovalent binder. 

For example, Zaidman et al. developed *Covalentizer*, an automated pipeline for the conversion of noncovalent binders to irreversible ones, which was successfully applied to the conversion of a SARS-CoV M^pro^ reversible inhibitor to a sub-micromolar SARS-CoV-2 M^pro^ irreversible one [373]. 

Valiente et al. reported the discovery of D-peptides that bind the spike RBD with low nanomolar affinity, hence blocking SARS-CoV-2 infection in cell-based assays. These ACE2-mimicking peptides were selected within the starting library through a combination of structural alignment, MD-based post docking refinement, and binding free energy estimation [374].

Similarly, a series of peptides mimicking the HR2 domain of the spike protein able to prevent SARS-CoV-2 infection in cell-based assays with low micromolar potency were designed through the combination between structural alignment, mutational scanning with the BeAtMuSiC [375] tool, and MD-based postdocking refinement [376]. 

Jeong et al. used Rosetta [377] to rationally design a mAb that recognizes a conserved surface on the spike RBD of various coronaviruses with picomolar binding affinities, thereby strongly inhibiting SARS-CoV-2 replication in cell-based assay [378]. 

A similar strategy was exploited by Miao et al., which employed Rosetta docking and MD-based postdocking refinement to design an RNA aptamer that binds with picomolar affinity to the spike RBD and inhibits SARS-CoV-2 replication with sub-micromolar potency in cell-based assay [379]. 

Further, Cao et al. utilized a combination of modeling with Rosetta and docking with RifDock [380] to design ten mini proteins which bind with picomolar affinity to the spike RBD thus inhibiting SARS-CoV-2 infection within cell-based assays [381]. 

Moreover, Glasgow et al. combined modeling with Rosetta and computational alanine scanning with Robetta [382,383] to rationally design “ACE2 receptor traps”, i.e., engineered proteins that bind the spike RBD with high affinity and neutralize SARS-CoV-2 infection as effectively as clinically used mAbs [384]. 

As thoroughly discussed in previous paragraphs, many SARS-CoV-2 drug discovery campaigns favored static, time-independent approaches such as docking or structural alignment, over time-dependent methods such as molecular dynamics. This can be attributed to the long calculation times, the reduced conformational sampling capabilities, and the lower accessibility of MD simulations to the general medicinal chemistry audience [331,385]. Despite these issues, several works demonstrated the potential of using full-fledged MD-based drug discovery pipelines, especially when smart enhanced-sampling strategies are employed [385]. 

For example, Bissaro et al. showed how high-throughput supervised molecular dynamics (HT-SuMD) [386], a virtual screening platform based on an enhanced sampling MD protocol, could be successfully exploited for docking fragments to the active site of SARS-CoV-2 M^pro^, overcoming accuracy limitations of most docking protocols [387] in identifying the native-like binding mode for frag-like compounds [388]. 

**Figure 5:**
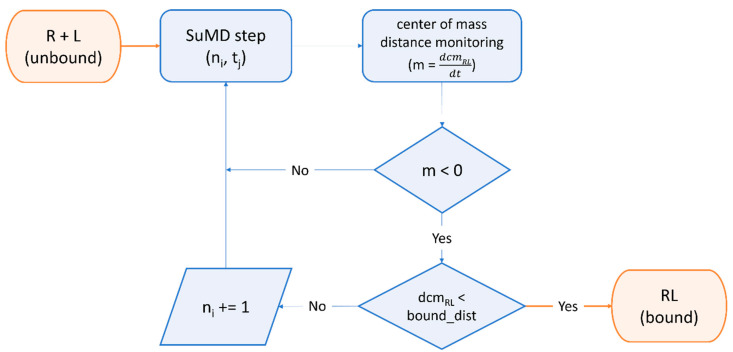
Workflow of a Supervised Molecular Dynamics (SuMD) simulation. The ligand is dynamically docked within a user-defined binding site through a series of short, unbiased molecular dynamics simulations. At the end of each step, the distance of mass between the ligand and the receptor binding site is computed for each trajectory frame and is fed to a tabu-like algorithm. If the slope of the straight line that interpolates the data is negative, indicating the ligand is approaching the binding site, the step is retained, and the simulation continues with the next “SuMD-step”. If not, the step is discarded and repeated, randomly reassigning particles’ velocities through the Langevin thermostat. This cycle is repeated until a threshold distance is reached or other user-defined termination criteria are met.

Furthermore, the SuMD [389,390] algorithm (Figure 5) was successfully exploited by Pavan et al. to decipher details about the recognition mechanism of Nirmatrelvir upon the SARS-CoV-2 M^pro^ catalytic site before any structural detail was revealed by the drug developer, with successive structural [189] and molecular medicine [198] studies confirming the prediction validity [391].

Moreover, an evolved version of the SuMD protocol was developed by Pavan et al. and successfully applied to the study of the recognition mechanism between RNA aptamers and proteins, including an RNA-aptamer that binds to the spike RBD with picomolar affinity thus preventing the viral infection of host cells [392]. 

## 3. Conclusions and Future Perspectives

Despite an unprecedented vaccination effort, which brought at least one vaccine shot to 70% of the world’s population [101,393], the battle against COVID-19 is far from won. Indeed, there is still a huge disparity between vaccination rates across first-world and low-income countries [101,393]. Furthermore, aside from the vaccines’ availability, several cultural and sociological factors contribute to the worldwide asymmetric vaccination coverage [394,395]. Finally, even in countries with the highest vaccination rates, the continuous emergence of novel viral variants [396] with enhanced immune escape capability sustains the viral spread even among the vaccinated population [397], so that to date a hundred thousand new COVID-19 cases are reported each day, leading, on an average, to a daily toll of hundreds of deaths globally [398]. Although the task of predicting the insurgence of novel variants of concern is not trivial [399], and the debate on the mechanism behind the genesis of these viral variants is still heated [400], it is reasonable to assume, based on the history of COVID-19 so far and other virus-related illnesses such as flu [401,402], that this phenomenon will continue to occur at least into the near future, forcing the scientific community to adapt existing treatments to emerging viral strains, other than developing novel therapeutics complementary to the existing ones [403]. Moreover, even if massive vaccination sensibly lowered the harmful effect on patients’ health caused by acute infection, long-term consequences of COVID-19 infections can still manifest at later stages [404], further reaffirming the need for tools that can effectively treat the disease other than preventing it. 

In conclusion, the take-home message from the present pandemic situation is that, among the strategies for identifying new therapeutic classes, with timescales compatible with those marked as a health emergency caused by a shapeshifting pathogen, the integration of structural biology information and new computational approaches probably represents the most promising one. The abundant amount of information provided by structural biologists coupled with the good predictive power of established computational workflows provides a quick platform for finding temporary solutions in the form of drug repurposing, allowing necessary time to develop more specific and tailored therapeutic entities. Although this strategy is not always successful in promoting hit compounds for clinical use [405], it can serve as a rational hypothesis generator for clinical studies, identify molecules to use as pharmacological tools to expand the knowledge on the etiopathogenesis of an emerging illness, and set the basis for the development of derivatives that can overcome the limitations of first-generation hits.

Despite all the scientific advancements in the field of computer-aided drug discovery, indeed, the time required for the release to the market of a new drug has not been sensibly reduced. Indeed, as highlighted in the work of Gupta et al. [406], many active compounds identified through structure-based drug design and computational techniques possess comparable activity to compounds in clinical trials. Many of these compounds, however, despite showing good antiviral activity and having a well-defined mechanism of action, fail to survive clinical stages of experimentation, due to the lack of good pharmacokinetic properties, which are essential for ensuring both good therapeutic efficacy and lack of intolerable side effects.

This fact further stresses the necessity for developing novel and complementary tools to the existing ones, especially in the evaluation of pharmacokinetic properties and off-target effects, which are usually the main causes of failure for candidate drugs in the clinical stages of experimentation. Accordingly, because the presented in silico approaches serve to provide candidates for preliminary selection, to extract the most value from these tools, predictions generated from computational approaches must be verified with biological confirmation, with both in vitro and in vivo models. Furthermore, with the increasing amount of curated experimental datasets becoming available to the scientific community, physics-based methods will be flanked more and more by artificial intelligence methods, both for evaluating the pharmacodynamic and pharmacokinetic properties of investigated compounds [407,408].

Finally, as estimated by a recent study [409], the likelihood of a highly infectious disease epidemic could double in the coming decades, indicating that the successful computational strategies applied in the biology domain that have been adopted against COVID-19 will most likely come in handy soon, providing us with robust and efficient solutions in tackling challenging diseases including new pandemics. 

## Data Availability

Not applicable.
